# Retraction Note: Flavonoids modulate multidrug resistance through wnt signaling in P-glycoprotein overexpressing cell lines

**DOI:** 10.1186/s12885-021-07827-3

**Published:** 2021-02-11

**Authors:** S. Mohana, M. Ganesan, N. Rajendra Prasad, D. Ananthakrishnan, D. Velmurugan

**Affiliations:** 1 Department of Biochemistry and Biotechnology, Annamalai University, Annamalai Nagar, Tamil Nadu 608 002 India; 2 Bioinformatics Infrastructure Facility (BIF), University of Madras, Guindy Campus, Chennai, Tamil Nadu India; 3 CAS in Crystallography and Biophysics, University of Madras, Guindy Campus, Chennai, Tamil Nadu India

**Retraction Note: BMC Cancer 18, 1168 (2018)**

**https://doi.org/10.1186/s12885-018-5103-1**

The editor has retracted this article [1]. Concerns were raised regarding a number of figures, specifically:

- Figure 2: the FZD1 blots in panels e (Rutin experiment) and f (Epicatechin experiment) appear to be identical.
- Figure 4b and c appear to be identical.
- Figure 4d and f appear to be very similar.
- Figure 4i and l appear to be very similar.

The data reported in this article are therefore unreliable.

M. Ganesan, N. Rajendra Prasad, D. Ananthakrishan and D. Velmurugan agree with this retraction. S. Mohana has not responded to correspondence regarding this retraction.
